# The impact of organisational change and fiscal restraint on organisational culture

**DOI:** 10.1186/s13033-016-0116-0

**Published:** 2017-01-13

**Authors:** Frances Dark, Harvey Whiteford, Neal M. Ashkanasy, Carol Harvey, Meredith Harris, David Crompton, Ellie Newman

**Affiliations:** 1 Metro South Mental Health District, 519 Kessels Road, Macgregor, QLD 4109 Australia; 2 School Public Health, The University of Queensland, Brisbane, Australia; 3 Policy and Epidemiology Group, Queensland Centre for Mental Health Research, Wacol, QLD Australia; 4 UQ Business School, The University of Queensland, Brisbane, Australia; 5 University of Melbourne, Melbourne, Australia; 6 North Western Mental Health, Melbourne, Australia; 7 Mobile Intensive Rehabilitation Team, Metro South Mental Health District, 519 Kessels Road, Macgregor, QLD 4109 Australia

**Keywords:** Organisational change, Organisational culture, Evidenced based practice, Fiscal restraint

## Abstract

**Background:**

Strategies to implement evidence-based practice have highlighted the bidirectional relationship of organisational change on organisational culture. The present study examined changes in perceptions of organisational culture in two community mental health services implementing cognitive therapies into routine psychosis care over 3 years. During the time of the study there were a number of shared planned and unplanned changes that the mental health services had to accommodate. One service, Metro South, had the additional challenge of embarking on a major organisational restructure.

**Methods:**

A survey of organisational culture was administered to clinical staff of each service at yearly intervals over the 3 years.

**Results:**

At baseline assessment there was no significant difference between the two services in organisational culture. At the midpoint assessment, which was conducted at the time the Metro South restructure was operationalized, there were less positive ratings of organisational culture recorded in Metro South compared to the other service. Organisational culture returned to near-baseline levels at endpoint assessment.

**Conclusions:**

These findings are consistent with the literature that organisational culture is relatively robust and resilient. It is also consistent with the literature that, at any one time, a service or organisation may have a finite capacity to absorb change. Consequently this limitation needs to be taken into account in the timing and planning of major service reform where possible. The results also extend the literature, insofar as external factors with a high impact on the operation of an organisation may impact upon organisational culture albeit temporarily.

## Background

Health service organisations are coming under tighter management to contain costs and improve outcomes [1–3]. Fiscal restraint, where growth in health expenditure is controlled to stay within budget, is a reality that is viewed as not necessarily inconsistent with delivering quality health care [4]. One strategy to achieve this is by implementing evidence-based practice (EBP). Dissemination and implementation of innovations (as would be necessary to introduce EBP in a health setting) require attention to external organisational factors (e.g. political, social, and economic factors) and internal organisational factors (e.g., culture, climate, and workforce skills) [5].There is also a recognition that these contextual variables operate at multiple levels and interact in a bidirectional way [6]. Ideally, health policy and funding work in concert with internal organisational processes to improve system performance and health care outcomes. In practice, however, health service organisations often have to absorb unplanned challenges while undergoing the change necessary to introduce EBP.

The ability of an organisation to incorporate change depends, in part, on its organisational culture [7]. Organisational culture, in this study, refers to the underlying values, and shared assumptions that influence behaviour within an organisation and is taught to new members. [8]. Proposed change dissonant to the prevailing organisational culture can present barriers or cause innovation to fail [9]. To facilitate innovation, the literature points to the need to address the so called implementation drivers of organisational systems, workforce competence and capability and leadership [9]. Some organisational culture measures tap into related domains of leadership, communication, planning and perceived support of the work force [10]. Implementation of system change occurs in stages and over time. It can be difficult to predict and to account for emergent challenges over the implementation period [9, 11]. Planned change management processes will often take organisational culture into account, with attention to ensuring buy-in from staff to assist staged change [12]. Unplanned change, especially that arising from external forces, combined with limited organisational control and limited ability for proactive planning, can adversely impact organisational culture [2, 9].

In the last 30 years there has been a focus on organisational culture as a possible malleable factor to address organisational performance [13]. It has been suggested that organisational culture in mental health services influence work attitudes, staff retention and work performance [14]. In addition, organisational culture has been shown to influence the implementation of EBP in mental health services [15]. Despite this interest in organisational culture, there seems to be little evidence of an effective strategy to improve organisational culture [2].

The present study sought to examine changes in staff perceptions of organisational culture assessed at yearly intervals over a 3 year period in two public mental health services in Brisbane, Australia: Metro South Mental Health Service and Metro North Mental Health Service. The observation period corresponded to a number of planned and unplanned system changes (Table 1).

**Table 1:** Timeline of external organisational context changes occurring during the study period

Date	Issue
March 2012	State Government change
July 2012	Health change from centralised organisation to local health networks
September 2012	Health budget cuts with the loss of 2754 staff
January 2013	Operationalization of the restructure of Metro South Mental Health Service commences
June 2013	Federal Government change
July 2013	Commission of enquiry report on the payroll dispute
November 2013–April 2014	Doctors contract dispute

### Organisational context

Organisational context refers to the broad array of circumstances and characteristics operating within and upon a service system [5]. A further distinction can be made between internal organisational context, and external organisational context.

#### Internal organisational context

The internal organisational context includes the structural aspects of the organisation as well as features of the workforce. Metropolitan health services in Brisbane comprise two sectors: Metro South and Metro North, with similar population, geographic, demographic features and socioeconomic profile. The Metro South Mental Health service serves a population of around 920,000, of which three thousand people have severe and persistent mental illness with complex needs. The area has high rates of socioeconomic disadvantage with 19.8% of people postponing mental health care because of cost [16]. At the time of the present study, Metro North had a population of around 900,000 with a similar demographic profile [17]. The sector includes 12.5% of the population who are disadvantaged. There are also subregions of Metro North with particular disadvantage, poorer health outcomes and low socioeconomic status. The geographic areas cover inner metropolitan suburbs to semi-rural areas in both service districts.

Metro North Mental Health Service and Metro South Mental Health Service have developed different strategies to address issues of inequitable distribution of resources. In 2012, the Metro South Mental Health service reorganised along diagnostic/model of services lines. Seven academic clinical units were established: Rehabilitation, Psychosis, Mood, Aged care, Consultation/Liaison, Resource and Access and Child and Youth [18]. This was an innovative organisational change aimed at addressing systemic issues in quality of service provision and optimal resource allocation. The aim of the restructure was to facilitate the adoption of EBP and to ensure more equitable distribution of resources throughout the service.

#### External organisational context

The external organisational context refers to the factors operating outside the service; and includes the broader socio-political and economic circumstances influencing the operation of the service. During the period of implementation, a number of unpredicted external factors emerged, affecting both services; these were driven by decisions at the state and federal levels of government (Table 1). The Australian health system involves three layers of government. The Commonwealth government is responsible for the universal medical and pharmaceutical benefits schemes, the cost of which is met in part by national health insurance. The State and Territory governments are primarily responsible for public health services including public hospitals. Local governments provide environmental health and healthy lifestyle programs.

This is a complex interdependent system posing challenges for health reform [4]. About half of the funding the State and Territory governments use to fund their services come from the Commonwealth government through grants and changes in Commonwealth Government priorities and funding influence the capacity of the State governments to deliver their health services.

Within the 3-year period of this study, a new Queensland state government was elected (in March 2012). Health services were reorganised to establish local area health networks overseen by boards. The state health budget was reduced with the loss of 2754 staff. There were reductions in the Commonwealth Health Budget following the change of government in 2013 (Australian budget 2014–2015) after which the Queensland Health budget was reduced by an estimated two billion dollars [19].

The health system is increasingly turning to technology to provide data to guide policy decisions but also to improve service management. In Queensland, an attempt to upgrade the payroll system failed; this impacted staff payments and led to a Commission of Inquiry in July 2013. Another protracted dispute occurred (November 2013–April 2014) with an attempt to change public sector medical staff contracts resulting in resignation of medical staff from services.

## Aim

The present study sought to explore perceptions of organisational culture in two similar mental health services over a 3 year period.

### Hypotheses

1. There would be no significant difference in clinical staff perceptions of organisational culture between Metro South Mental Health Service and Metro North Mental Health Service at baseline.
2. At midpoint assessment, Metro South Mental Health Service would have lower scores on the measure of organisational culture compared to Metro North Mental Health Service because of the added impact of organisational restructure.
3. There would be no significant difference in staff perceptions of organisational culture in Metro South Mental Health Service and Metro North Mental Health Service at endpoint assessment because of the relative resilience of organisational culture to change.

## Methods

### Design

This study used a repeated cross-sectional survey design at three annual time points in Metro South Mental Health Service and Metro North Mental Health Service, in Brisbane.

### Participants

Study participants comprised 105 staff members from community teams of the Metro South Mental Health Service (*N* = 54) and Metro North Mental Health Service (*N* = 51). The mean age of staff at baseline was 39.73 years (*SD* 9.20 years), at midpoint assessment was 43.13 years (*SD* 10.50 years) and endpoint was 41.37 (*SD* 9.83 years) (Table 2).

**Table 2:** Characteristics of participants at each survey occasion

Demographic variables	Baseline assessment		Mid assessment		End assessment	
	N	%	N	%	N	%
Site						
Metro south	25	46	51	94.4	35	64.8
Metro north	26	50.9	32	62.7	24	47.0
Sex						
Males	15	29.4	36	43.4	19	32.2
Females	36	70.6	47	56.6	40	67.8
Education						
Secondary	–	–	2	2.4	1	1.7
Technical college	3	5.9	1	1.2	1	1.7
Hospital	2	3.9	4	4.8	0	0
University	22	43.1	38	45.8	30	50.8
Post-graduate	24	47.1	37	44.6	27	45.8
Discipline						
Nursing	20	41.2	32	38.6	15	25.4
Allied health	12	23.5	24	28.9	19	32.2
Psychology	5	9.8	12	14.5	11	18.6
Psychiatry	8	15.7	15	18.1	8	13.6
Other	5	9.8	–	–	6	10.2
Role						
Peer support worker	–	–	–	–	5	8.5
Case manager	20	39.2	44	53.0	19	32.2
Clinician	4	7.8	–	–	3	5.1
Nurse	8	15.7	5	6.0	–	–
Occupational therapist	3	5.9	1	1.2	2	3.4
Psychologist	1	2.0	8	9.6	11	18.6
Psychiatrist	7	13.7	6	4.8	1	1.7
Registrar	–	–	4	7.2	3	5.1
Medical officer	–	–	1	1.2	–	–
Team leader	1	1	8	9.6	9	15.3
Professional lead	1	1	1	1.2	1	1.7
Other	4	3.9	5	6.0	5	8.5

Baseline N = 51, Mid Assessment N = 83, End Assessment N = 59

### Sampling procedure

At each time point (March–April 2013, 2014, 2015), all members of the clinical staff population were identified by the team leaders of each team. The baseline survey was distributed to all clinical staff in hard copy with written consent obtained. At mid- and endpoint assessment, the surveys were distributed via a link sent in an email to potential participants and completed online. Informed consent was implied by the completion of the (non-identified) survey. The survey was open to receive replies for one month at each time point.

### Measures

The following information was collected via the surveys administered at each time point:

Socio-demographic characteristics (e.g. age, sex and education) and job classification (e.g. discipline and role) was collected. Staff perception of organisational culture was measured using the Organisational Culture Profile (OCP: [20]). The OCP is a 65-item questionnaire used to measure an individual’s perception of organisational culture tapping into 10 domains; leadership, structure, innovation, job performance, planning, communication, environment, humanistic workplace, development of the individual and socialisation on entry [10]. The instrument is constructed using a 7-point Likert-type scale with 1 being “strong disagreement” and 7 being “strong agreement”. Analysis of the psychometric properties of the OCP in the Australian regional healthcare sector found the domains of leadership, planning, communication and humanistic workplace as reliable dimensions (α > .80) [1]. Thus, the present study sought to examine the reliable domains of leadership, planning, communication and humanistic workplace; and excluded job performance, and development of the individual from the analyses.

### Statistical analyses

Data were analysed using Statistical Package for Social Sciences (SPSS) version 20. Independent single sample *t*-tests were used to compare staff perceptions of organisational culture in Metro South Mental Health Service compared to Metro North Mental Health Service at each of the three time points (baseline, midpoint and endpoint). *p* values <.05 were considered statistically significant.

## Results

### Response rates

The staffing profile of respondents at the three time-points is provided in Table 2. Fifty-one staff (48.3%) responded to the survey at baseline, 83 (79%) at mid-assessment and 59 (56.2%) at end-assessment. A greater percentage of respondents came from Metro South Mental Health Service at midpoint (61% Metro South Mental Health Service, 38.6% Metro North Mental Health Service) and end-assessment (59% Metro South Mental Health Service, 40.7% Metro North Mental Health Service) (Table 2). The majority of respondents had university or post graduate qualifications. Over 50% of the participants were female across the three time-points. A typical rank ordering of staffing profiles for Metro South Mental Health Service and Metro North Mental Health Service is 50% nursing staff, 30% allied health, 10% psychologist and 10% medical. Although the rank order of disciplines responding reflected this staffing profile at baseline and midpoint, there were relatively fewer responses from nurses at endpoint. The most commonly endorsed role was “case manager” which reflects the dominant tasks required of community mental health workers in the services studied.

### Comparing Metro North and Metro South

There was no significant difference between Metro South Mental Health Service and Metro North Mental Health Service on any of the four included OCP domain scores at baseline (Table 3). At midpoint, significantly lower scores were observed in leadership (*t* [81] = 3.73, *p* = .0001), planning (*t* [81] = 2.72, *p* = .008), communication (*t* [81] = 2.68, *p* = .009) and humanistic (*t* [81] = 2.36, *p* = .026) domains in Metro South Mental Health Service compared to Metro North Mental Health Service (Table 3). No significant differences between Metro South Mental Health Service and Metro North Mental Health Service in OCP scores were found at endpoint assessment (Table 3).

**Table 3:** Means and standard deviations of organisational culture domains across assessment points

Assessment point	Site	Organisational culture domain	Mean	SD
*Baseline assessment*	Metro North *N* = 26	Leadership	4.92	1.34
		Planning	5.00	1.28
		Communication	4.54	1.31
		Humanistic	4.70	.73
	Metro South *N* = 25	Leadership	4.96	1.12
		Planning	4.78	.92
		Communication	4.51	1.19
		Humanistic	4.81	1.05
*Mid assessment*	Metro North *N* = 32	Leadership	5.15	1.07
		Planning	5.00	1.20
		Communication	4.60	1.36
		Humanistic	4.68	1.08
	Metro South *N* = 51	Leadership	4.08	1.37
		Planning	4.24	1.32
		Communication	3.76	1.39
		Humanistic	4.09	1.20
*End assessment*	Metro North *N* = 24	Leadership	5.19	1.12
		Planning	4.94	.98
		Communication	4.44	.97
		Humanistic	4.56	1.09
	Metro South *N* = 35	Leadership	5.16	1.20
		Planning	5.12	1.05
		Communication	4.42	1.15
		Humanistic	4.74	.92

## Discussion

The present study sought to explore organisational culture in two mental health services over a 3 year period during which time various planned and unplanned system changes occurred. Organisational culture is reported to be relatively robust and difficult to alter [3]. Although mental health systems are renowned for being complex systems that frequently need to absorb challenges, the confluence of forces impacting on mental health services in Queensland in the period of 2013–2015 was unusual and severe, impacting on staffing levels (redundancies) and staff morale (payroll dispute and Doctors contract dispute) (Table 1).

Consistent with the first hypothesis, there were no significant differences between the organisational culture of each service at baseline.

Consistent with the second hypothesis, we found that Metro South Mental Health Service had lower scores across all four included organisational culture domains at mid-assessment compared to Metro North. This research cannot determine causal links for the change in measures of organisational culture but the change occurred at a time corresponding with the impact of additional fiscal restraint and the initiation of structural changes specific to that service during 2013.

The implementation literature refers to absorptive capacity of organisations—inferring that there is a threshold above which adverse effects may occur or context which may not be favourable to implementation [21]. The added stressor of unplanned changes and planned-but -major organisational restructure may have exceeded the absorptive capacity of Metro South Mental Health Service as reflected in staff perceptions of organisation culture at midpoint assessment.

The third hypothesis was also supported, with recovery in organisational culture as measured by the OCP at endpoint being consistent with the reported “elastic” nature of organisational culture [8]. In addition, the extraordinary and unplanned nature of some of the stressors may have created a crisis that enabled positive and relatively rapid organisational change that may have been difficult to enact in usual circumstances.

Current health services have incorporated modern organisational processes and regularly plan and use change management principles to innovate and improve [21]. The Metro South Mental Health Service had been planning a major service restructure for around a year and was committed to change when the external factors of political change and reorganisation of health from centralised to a decentralised structure and budget cuts occurred.

The results of the present study are consistent with the literature [6] and, as a whole, the staff perceptions of organisational culture within mental health services declined temporarily at a time corresponding to the organisational impact of system changes but returned to baseline levels within a relatively short period of time.

### Impact of restructure and fiscal restraints

In Metro South Mental Health Service, where there had been the additional change of internal organisational restructure, the organisational culture deteriorated before rebounding in the 3rd year of the study. These results appear to support the robustness of organisational culture as described in the literature [3]. It is not possible to disentangle whether the internal reorganisation of the service influenced the return of the organisational culture to baseline levels or whether the changes alone have made the organisational culture stronger.

The organisational structural changes in Metro South Mental Health Service were aimed in part at facilitating EBP and required a collective shift in work behaviour that can be perceived as a burden by staff [22]. An extended process of consultation helped prepare staff for these internal changes. This planning may have enabled greater accommodation of change. The maturation of the functioning of these supporting structures in the 3rd year may have influenced the more positive endorsement of organisational culture in the last survey.

### Limitations

As with all research, the present study suffers from limitations; we identify five of these. First, the study is limited by its cross-sectional design, thus making causality difficult to ascertain. Second, although the described unplanned or planned system change was major, other factors not taken into account may have been influential in the results and therefore it is not possible to know whether these described changes were the cause of the observed results. Third, the OCP questionnaire was part of a larger survey examining the implementation of cognitive therapies within mental health services and may have influenced the responses of those completing with “survey fatigue” and constraint of perceived context to that of cognitive therapies rather than the organisation as a whole. Fourth, the primary author was the principal investigator of the study and a Director within Metro South Mental Health Service; thus, while participant responses were de-identified this may have influenced the way in which staff members reported their perceptions of organisational culture. Fifth, the small sample size limited power to detect differences. The low response rate at baseline and midpoint may mean our results are not representative of the whole population (Table 2).

### Recommendation for future research and conclusions

Future research should consider alternative research designs, such as repeated measures, to establish causal links and trends over time. Where data collection is compromised by a participant researcher in a position of power within an organisation, consideration needs to be given to having an objective research team not working within the service being studied.

Surveying a larger population such as all state based mental health services and providing incentive for completed and returned forms would have increased the samples size and return rates enabling more confident predictions about the organisational culture of the health services surveyed.

Overall, our findings suggest that staff perceptions of organisational culture are relatively elastic and organisational culture is able to absorb some change. Any major service reform can and should be well planned as external forces, such as fiscal restraint impinging on the system can be unpredictable and influence successful implementation of change.

The concept of path dependence where the possibility for change is influenced by the history of the organisation is increasingly being recognised as a factor in health reform [23]. The path dependency theory would be a useful paradigm for any future research.

## Abbreviations

EBP: evidence-based practice
OCP: Organisational Culture Profile
SPSS: statistical package for social sciences
